# Immunotherapy for HER-2 positive breast cancer

**DOI:** 10.3389/fonc.2023.1097983

**Published:** 2023-03-16

**Authors:** Tingting Yang, Lihua Kang, Dan Li, Yanqiu Song

**Affiliations:** Cancer Center, The First Hospital of Jilin University, Changchun, Jilin, China

**Keywords:** breast cancer, HER2+, passive immunotherapy, immune checkpoint inhibitors, adoptive T-cell immunotherapies, active immunotherapy

## Abstract

Immunotherapy is a developing treatment for advanced breast cancer. Immunotherapy has clinical significance for the treatment of triple-negative breast cancers and human epidermal growth factor receptor-2 positive (HER2+) breast cancers. As a proved effective passive immunotherapy, clinical application of the monoclonal antibodies trastuzumab, pertuzumab and T-DM1 (ado-trastuzumab emtansine) has significantly improved the survival of patients with HER2+ breast cancers. Immune checkpoint inhibitors that block programmed death receptor-1 and its ligand (PD-1/PD-L1) have also shown benefits for breast cancer in various clinical trials. Adoptive T-cell immunotherapies and tumor vaccines are emerging as novel approaches to treating breast cancer, but require further study. This article reviews recent advances in immunotherapy for HER2+ breast cancers.

## 1 Introduction

Breast cancer is the most common cancer among women worldwide, meanwhile,it is a heterogeneous disease and is associated with its different tumor microenvironment. Human epidermal growth factor receptor-2 positive (HER2+) breast cancers are aggressive and associated with poor prognosis. HER2 is amplified or overexpressed in an estimated 20%-25% of breast cancers (1). HER2+ was defined by immunohistochemistry (IHC) score 2+/3+ or fluorescence *in situ* hybridization (FISH) positive. IHC detects HER2 protein overexpression using monoclonal or polyclonal antibodies which based on subjective determination of the intensity of the color reaction. FISH detects by fluorescence microscopy in different samples, such as cell lines, frozen tissue, paraffin embedded tissue and micro-tissue arrays, with the principles of *in situ* hybridization: DNA probes complementary togenomic sequences of interest are generated, labeled and then hybridized to the target tissue (2).

Multiple genes have been detected co-amplified with HER2 after identification of HER2 amplification in breast cancer. In one study (3), 2 Mb region at the 17q12eq21 using FISH and RTPCR identified a common region of amplification in primary breast tumors which including six genes amplified and overexpressed (HER2, GRB7, PNMT, MLN64, MGC9753, and MGC14832). Sahlberg KK.et identified a minimal common region of amplication (MCR) including 6 genes covering 78.61 Kb (STARD3, TCAP, PNMT, PERLD1, HER2, and C17orf37) by aCGH analysis, showing that nearly all tumors amplify a considerably larger region, 1.74 Mbp on average, suggesting that many additional genes in the region around HER2 are also amplified and may contribute to the cancer phenotype (4).

Cancer immunotherapy can trigger anti-tumor response *via* regulating the immune microenvironment. Immunotherapy (e.g. immune checkpoint inhibitors) has shown anti-tumor activity in a variety of cancers, such as melanoma and prostate cancer (5–7). Immunotherapy has been applied to triple negative breast cancers (TNBCs) (8) as well, but the efficacy of immunotherapy for HER2+ breast cancers remains unclear and requires further investigation.

Breast cancer has traditionally been considered an immunologically “cold” tumor with a low tumor mutational burden (TMB) (9) and unresponsive to immunotherapy. However, breast cancer is heterogeneous, and some subtypes of breast cancer exhibit certain indicators for immunogenicity, including TMB, high tumor infiltrating lymphocytes (TILs), and expression of immuno-inhibitory molecules, which may guide selection of immunotherapy. HER2+ breast cancers and TNBCs share similarities in TMB, TILs and PD-L1 expression; therefore, these patient populations are proposed to have significant benefit from immunotherapy. In an analysis of breast cancer samples from the Breast International Group (BIG) 02-98 phase III adjuvant trial with operable, clinical stage T1 to T3 invasive breast adenocarcinoma, with at least one positive axillary lymph node, HER2+ breast cancers and TNBCs were significantly more likely to be infiltrated by higher numbers of TILs than estrogen receptor positive (ER+) or HER2-negative (HER2-) breast cancers; HER2+ breast cancers had a higher TMD compared to luminal tumors; and TILs were considered predictive biomarkers for cancer immunogenicity (10, 11).It is well known that the molecular heterogeneity of the tumor itself and the heterogeneity of the tumor microenvironment affect the therapeutic response of anti-HER2 agents. Immune invasion may be clinically relevant and heterogeneous in specific molecular breast cancer subtypes, with stromal cell/protein composition that varies significantly between patients and tumor stage. Immune cells within the tumor microenvironment(TME)actively interact with cancer cells but also each other, shaping the multi-directional crosstalk that evolves continuously during tumor progression. Immune cells within the tumor microenvironment (TME) interact with cancer cells, but also with each other, forming complex factors that influence the process of tumor progression. TME presents what is defined by different cellular and non-cellular components(i.e., ECMs) in the tumor stroma.(Impact of Immune Cell Heterogeneity on HER2+ Breast Cancer Prognosis and Response to Therapy )

Currently, immunotherapy for HER2+ breast cancers can be divided into four categories according to the mechanism of action: (1) passive immunotherapy, including monoclonal antibodies; (2) immune checkpoint inhibitors, including programmed death receptor-1 (PD-1)/programmed death ligand 1 (PD-L1) inhibitors; (3) adoptive cell immunotherapy, including chimeric antigen receptor (CAR)-T cell therapy; and (4) active immunotherapy with vaccines.

## 2 Passive Immunotherapy: Antibody-dependent cytotoxicity

Antibody-dependent cytotoxicity (ADCC) is an adaptive immune response through which Fc receptor-bearing effector cells lyse antibody-coated target cells that express pathogen-derived antigens on their surface (12).

### 2.1 Monoclonal antibodies

Monoclonal antibodies engage natural killer cells to target tumors using ADCC (13),

which is mediated by effector immune cells, particularly CD56dimCD16+ NK cells (14, 15).

Trastuzumab and pertuzumab are HER2-targeted recombinant humanized monoclonal antibodies. Trastuzumab blocks homodimerization of HER2 by binding its ectodomain. Pertuzumab blocks heterodimerization between HER2 and other ErbB receptors (especially HER3) by binding to HER2 subdomain II (16). Trastuzumab and pertuzumab have complementary antitumor mechanisms of action. They can mediate ADCC, inhibit downstream signaling (such as MAPK signaling), and/or increase endocytosis of the HER2 receptor (17). The simultaneous binding of pertuzumab and trastuzumab to HER2 increases the density of Fcγreceptor (FCGR) binding sites on HER2+ tumors (18). The binding of FCGR to trastuzumab Fc region initiates the ADCC process, resulting in the secretion of perforin and granzymes by immune effector cells, which trigger apoptosis of tumor cells by inducing caspase activity and DNA fragmentation (14, 19, 20).

Initially in mouse model studies, the key role of host Fc receptors in mediating antitumor activity within trastuzumab antibodies was shown, followed by a broader range of immune activity, including innate and adaptive immune function, memory, and cytokines (21–24).

Studies indicated that the interaction of anti-HER2 antibodies with FCGRs expressed on innate immune cells, such as monocytes,macrophages, natural killer (NK) cells, and dendritic cells may be involved in their therapeutic activity (25).

Interaction between antibody Fc fragment and FcgRs eliminates antibody-bound tumor cells *via* ADCC mainly by NK cells, or *via* antibody-dependent cellular phagocytosis (ADCP) mainly by macrophages (21, 26). Studies have shown that ADCP is mainly regulated by antiphagocytic “don’t eat me” signals that are amplified in many cancers (27).;Chief among these is CD47, which has been demonstrated to be upregulated in BC, bind to and trigger signaling of macrophage SIRPα to suppressing phagocytosis (28, 29). The study of HER2/SIRPα (CD47) dual antibody suggests that CD47 antibody mediates the anti-tumor effect of ADCP to enhance HER2 antibody. By using NK cell deficient mice, there is no evidence for NK cell involvement in Trasruzumab function. ADCP activity can be enhanced through strategies that inhibit tumor growth and potentially enhance antigen presentation, which may be critical to overcome resistance to HER2 monoclonal antibody therapy (30).

#### 2.1.1 Trastuzumab, pertuzumab, and HLX02

Dual HER2 therapy combining trastuzumab and pertuzumab with chemotherapy is standard first-line therapy for most advanced breast cancer patients and for neo/adjuvant therapy in early stage breast cancer patients.

##### 2.1.1.1 Early Breast cancer setting

One-year adjuvant trastuzumab treatment significantly reduces recurrence and mortality associated with early stage HER2+ breast cancer (17). In the neoadjuvant setting, in the NeoSphere and PEONY trials, trastuzumab in combination with pertuzumab and chemotherapy showed significant improvements in total pathological complete response compared to controls in patients with HER2+ early or locally advanced, inflammatory breast cancer. These findings were supported by survival outcomes (progression-free survival [PFS] and disease-free survival [DFS]) (31, 32).

Trastuzumab and pertuzumab are usually administered intravenously for 1.5-2 hourstime long, which can represent a burden on patients and the healthcare system. This burden may be alleviated by a fixed-dose combination of pertuzumab and trastuzumab for subcutaneous injection that can be finished in 5-8 min. In the FeDeriCa trial, with the neoadjuvant, the fixed-dose combination of pertuzumab and trastuzumab by subcutaneous injection provided similar total pathological complete response rates to intravenous injection of pertuzumab plus trastuzumab in patients with early stage HER2+ breast cancer. Safety was consistent acceptable with other pertuzumab, trastuzumab, and chemotherapy trials.

In 2020, the US Food and Drug Administration (FDA) approved the fixed-dose combination (pertuzumab, trastuzumab, and hyaluronidase-zzxf) with chemotherapy as neoadjuvant therapy for patients with early or locally advanced, inflammatory HER2+ breast cancer, and for high-risk patients with early HER2+ breast cancer (33).

##### 2.1.1.2 Metastatic Breast cancer setting

CLEOPATRA was a phase 3 study aiming at comparing the efficacy and safety of pertuzumab, trastuzumab, and docetaxel with placebo, trastuzumab, and docetaxel in patients with HER2-positive metastatic breast cancer. The PFS and OS were significantly improved in the pertuzumab group compared with the placebo group (34). In MetaPHER, the largest study to evaluate the efficacy and safety of subcutaneous trastuzumab plus intravenous pertuzumab and chemotherapy in patients with HER2+ metastatic breast cancer, safety and efficacy were consistent with histological evidence of intravenous trastuzumab in this combination (35).

HLX02 as a trastuzumab biosimilar was developed to meet abroad patient need. In global clinical trials, HLX02 and trastuzumab were bioequivalent (36). HLX02 is recommended by the National Comprehensive Cancer Network (NCCN) guidelines for breast cancer and is approved for use in HER2+ early and metastatic breast cancer in Europe and China.

#### 2.1.2 Antibody⁃drug conjugates (ADC)

T-DM1(ado-trastuzumab emtansine) is the first ADC targeting the HER2 receptor. T-DM1 comprises trastuzumab conjugated with a cytotoxic moiety (DM1, derivative of maytansine) that inhibits microtubule assembly. T-DM1 enters cancer cells through HER2 receptor-mediated endocytosis.

##### 2.1.2.1 Early Breast cancer setting

Among patients with HER2-positive early breast cancer who had residual invasive disease after completion of neoadjuvant therapy, the risk of recurrence of invasive breast cancer or death was 50% lower with adjuvant T-DM1 than with trastuzumab alone(free of invasive disease at 3 years was 88.3% in the T-DM1 group and 77.0% in the trastuzumab group) in KATHERINE trial (37).

##### 2.1.2.2 Metastatic breast cancer setting

In the EMILIA trial, T⁃DM1 significantly prolonged PFS and overall survival (OS) with fewer adverse events compared to lapatinib plus capecitabine in patients previously treated with trastuzumab and a taxane for locally advanced or metastatic breast cancer (38). In the TH3RESA trial, T-DM1 significantly improved OS compared to previously treated with both trastuzumab and lapatinib (advanced setting) and a taxane (any setting for HER2+ advanced breast cancer) and with progression on two or more HER2-directed regimens in the advanced setting (39). The NCCN breast cancer guidelines recommend that T-DM1 is useful as preoperative/adjuvant therapy in certain circumstances in patients with HER2+ invasive breast cancer or as second-line treatment for recurrent unresectable (local or regional) or stage IV (M1) disease after pertuzumab, trastuzumab and docetaxel or paclitaxel (40).

DS-8201(T-DXd). It comprises a humanized anti-HER2 antibody, an enzymatically cleavable peptide linker, and an exatecan-derivative topoisomerase I inhibitor (DXd) (41). In the DESTINY-Breast 01 trial, DS-8201 had durable antitumor activity in patients with metastatic HER2+ breast cancer previously treated with T-DM1 (42). In the DESTINY-Breast 03 trial, there was a lower risk of disease progression or death with DS-8201 compared to T-DM1 in patients with HER2+ metastatic breast cancer pre- treated with trastuzumab and a taxane (43). In 2017, the FDA granted DS-8201 as breakthrough therapy for the patients with HER2+ locally advanced or metastatic breast cancer who had been treated with trastuzumab and pertuzumab and had disease progression after T-DM1. In 2019, the FDA accelerated approved DS-8201 for treating HER2+ unresectable or metastatic breast cancer who have been experienced two or more anti-HER2 treatment. In 2022, the FDA approved DS-8201 for unresectable or metastatic HER2+ breast cancer adult patients who had received a prior anti-HER2-based regimen in the metastatic setting or the neoadjuvant or adjuvant setting however, had developed disease recurrence during or within 6 months of completing therapy.

The key factors are linker chemistry, activity of cytotoxic payload molecules, properties of mAbs, payload-to-antibody ratio, and cell permeability and potency, all of which contribute to the observation of clinical activity.The key peculiarities of this T-DXd include HER2mAB and payload coupling *via* a cleavage linker; It has a high drug-antibody ratio of 8:1; The cytotoxic payload (deruxtecan, a highly potent topoisomerase I inhibitor) is membrane-soluble; And, once released in HER2+ cells, the molecule diffuses out of the cell and can produce cytotoxic effects on surrounding HER2 tumor cells and the TME, a feature defined as the “bystander effect” (43). T-Dxd has been shown to exceeded clinical activity of T-DM1 and activity against T-DM1-resistant diseases (44).

ADCs interact with cancer and immune cells, primarily through mechanisms such as induction of immunogenic cell death, antibody-dependent cell-mediated cytotoxicity, and dendritic cell activation, ultimately in potential synergy with immunotherapy. In fact, ADCs induce tumor-specific adaptive immunity and increase the invasion of T cells into the tumor microenvironment, while immune checkpoint inhibitors rejuvenate depleted T cells and enhance anti-tumor immune responses. Given promising preclinical data, clinical trials of multiple tumor types are currently underway to evaluate the safety and activity of the combination regimen (45).

T-DXd increased tumor-infiltrating DCs and upregulated the expression of their maturation and activation markers, increased tumor-filtered CD8+ T cells, and enhanced the expression of PD-L1 and MHC class I on tumor cells (45). Much of the mechanistic foundation for the broad clinical activities of these ADCs remains unknown and eagerly awaits new hypotheses and experimental studies*.*

### 2.2 Enhancing ADCC-mediated cytotoxicity

In ADCC, Fc-mediated binding of antibodies to CD16A on NK cells enables NK cells to lyse cells without priming and secrete cytokines to recruit adaptive immune cells (46).Several factors can influence the ability of HER2-directed therapies to trigger ADCC, such as Fcγ receptor polymorphisms and the quantity of TILs. Some strategies have been investigated to enhance ADCC associated with HER2-targeted therapy. CD16A, CD32A, and CD32B expressed on immune cells modulating ADCCs (30).

#### 2.2.1 Enhancing ADCC by altering the affinity of the antibody to FcγR

Margetuximab (MGAH22) is a chimeric monoclonal antibody that targets HER2. MGAH22 binds the same HER2 receptor epitope as trastuzumab. MGAH22 has increased binding affinity to CD16A (FcγRIIIa), which enhances NK cell-mediated ADCC, while reduced binding to CD32B which is an inhibitory FcγR. In the SOPHIA (47) trial, MGAH22 plus chemotherapy had a satisfactory safety profile and showed a significant improved PFS compared to trastuzumab plus chemotherapy in patients with HER2+ advanced breast cancer pre-treated with 2 or more anti-HER2 therapies.

### 2.3 Enhancing ADCC by alteration of glycosylation

Changes in glycosylation patterns can increase ADCC activity by increasing FcγR binding. Among the oligosaccharides in the Fc domain, fucose plays the greatest role in determining binding to FcγRIIIA. TrasGEX is a second -generation monoclonal antibody of trastuzumab. It has the same Her2 binding properties as trastuzumab, but has been glyco-optimized for enhanced ADCC. In a Phase I dose escalation study, TrasGEX was safe and well tolerated, and showed antitumor activity in 50% of evaluable patients with HER2+ solid tumors (48).

#### 2.3.1 Bispecific antibodies

Bispecific antibodies can simultaneously target two different antigens or epitopes of the same antigen and enhance ADCC. MCLA-128 is a bispecific humanized full-length IgG1 antibody targeting HER2/HER3. MCLA-128 inhibits HER2/HER3-driven cell growth and can overcome HER3-mediated resistance to HER2-or EGFR-targeted therapies. A phase II clinical trial is underway in evaluating MCLA-128 with trastuzumab/chemotherapy in HER2+ breast cancer and with endocrine therapy in ER+ and low HER2 breast cancer.

ZW25 is an anti-HER2 humanized bispecific antibody that binds two different epitopes in extracellular domains II and IV of HER2 (49). In a Phase I trial, ZW25 in combination with chemotherapy demonstrated antitumor activity in patients with unresectable locally advanced or metastatic HER2+ breast cancer pre-treated with trastuzumab, T-DM1, and pertuzumab (50).

[(HER2)2xCD16] is another bispecific antibody in a tribody format that can redirect CD16-expressing γδ T cells in addition to NK cells to lyse HER2-expressing tumor cells. In a preclinical study, [(HER2)2xCD16] was superior to trastuzumab in triggering γδ T cell and NK cell-mediated lysis of HER2-expressing cancers, such as pancreatic ductal adenocarcinoma breast cancer, and autologous primary ovarian tumor cells (51).

## 3 Immune checkpoint inhibitors

### 3.1 PD-1/PD-L1 inhibitors

PD-1 is a member of the CD28 superfamily. PD-1 widely expressed in tissues is an inhibitor of adaptive and innate immunity. PD-1 can attenuate T cell activation and cause immune tolerance (52). PD-L1 is upregulated in breast cancer, and the up-regulated expression is associated with clinicopathological features of breast cancer, such as large tumor size, high tumor grade, lack of hormone receptor expression, and HER-2 positivity (53). The cytotoxic effect of anti-HER2 targeted drugs is related to immunity. PD-1/PD-L1 inhibitors can enhance adaptive immunity. We look forward to the combination of the two strategies to further enhance the anti-tumor effect or reverse drug insensitivity and drug resistance. Based on the difference in immune response between early and late HER2 positive breast cancer, the reason may be that early patients have stronger immunity. At this time, the addition of PD-1/PD-L1 inhibitor can mobilize more effective CD8+T cells to play the ADCC effect, enhance the ability of auto immune recognition and killing, and thus fight against tumors. The anti-PD-1 pembrolizumab and nivolumab have been approved by the FDA for the treatment of metastatic melanoma. In 2019, PANACEA, phase1b-2 single arm multicenter study found that the level of TIL in PD-L1 positive group, objective response group and disease control group was significantly increased. Its research results showed that PD-L1 positive compared with PD-L1 negative patients with trastuzumab resistance and advanced HER2 positive breast cancer patients, the PD-1 inhibitor pabolizumab combined with trastuzumab could improve objective response rate(ORR) (15% vs. 0) and 1-year PFS (12% vs. 0), One-year OS (65% vs. 12%), and the clinical survival outcome is beneficial (54). However, more sample size and longer follow-up time are still needed to verify whether the combination of patients with moderate and high risk of recurrence in the early stage will improve ORR, prolong PFS and improve OS, which still needs to be confirmed by a large number of clinical trials.

Atezolizumab is another monoclonal antibody specific to PD-L1. Atezolizumab can re-activate suppressed T cells, and promote the activation of T cells in lymph nodes. Atezolizumab can disrupt the connections of PD-1-cytotoxic T cells, PD-L1-tumor cells, and B7-1-T cells (55),. In KATE2, T-DM1 plus atezolizumab did not show a clinically meaningful improvement in PFS compared to T-DM1 plus placebo in patients with HER2+ advanced breast cancer who have been treated with trastuzumab and a taxane (56). However, in a subgroup analysis, T-DM1 plus atezolizumab had a higher 1-year OS compared to T-DM1 plus placebo in patients with PD-L1+, HER2+ advanced breast cancer (94.3% vs. 87.9%, HR=0.55; 95% CI: 0.22-1.38). Biomarker evaluation showed higher expression of CD8 which is the marker of T cell activation, and more TILs in PD-L1+ tumors compared to PD-L1- tumors.

In the trial of Impassion 050, atezolizumab with neoadjuvant dose-dense doxorubicin/cyclophosphamide–paclitaxel and pertuzumab-trastuzumab did not increase pathologic complete response rates compared to placebo in patients with high-risk, HER2+early-stage breast cancer, including patients with PD-L1+ breast cancer (57). The overall safety profile was consistent with known profiles for atezolizumab in other combination studies. However, five fatal adverse events occurred (all in the atezolizumab group). Of these, two were considered treatment-related (alveolitis and septic shock) and two were attributed to COVID-19. The use of atezolizumab combined with a strong cytotoxic regimen requires careful patient monitoring and management of adverse events.

Anti-HER2-targeted drugs combined with immune checkpoint inhibitors are a promising new strategy for the treatment of HER2+ breast cancer. Preclinical studies show immunotherapy may enhance the efficacy of anti-HER2 therapy. In breast cancers, macrophages with PD-L1 and indolamine 2,3-dioxygenase (IDO) overexpression inhibited NK cell-mediated ADCC and T cell-mediated cytotoxicity after antibody-dependent cell phagocytosis (ADCP), thereby reducing the anti-tumor effects of monoclonal antibody therapy. In animal models, the combined application of inhibitors of PD-L1 and IDO significantly enhanced trastuzumab efficacy. Clinically, patients with HER2+ breast cancer had significantly increased PD-L1 and IDO expression in tumor-infiltrating macrophages after preoperative treatment with trastuzumab, which determined response to trastuzumab (58). At present, the researches of PD-1/PD-L1 inhibitor in HER2+ breast cancer are limited, and the sample size of the researches are small, and the evidences of evidence-based medicine are insufficient. The time of joining, the selection of population, the different clinical stages of cancer, the heterogeneity of tumor, the difference of individual immune response level, and the different immune microenvironment will affect the evaluation of efficacy, and further researches are needed. We summarized clinical trials assessing the combination of checkpoint inhibitors and HER-targeted treatment in HER2+ breast cancer in **Table 1**.

**Table 1:** Clinical trials assessing the combination of checkpoint inhibitors and HER-targeted treatment in HER2+ breast cancer.

Study	Phase	Setting	n	Treatment	Results/Status
PANACEA (55) (NCT02129556)	Phase Ib–II	Advanced	58	Trastuzumab+pembrolizumab	• ORR 6/40 (15%) in PD-L1+ pts • No responses among PD-L1− pts • Median PFS 2.7 m (90%CI 2.6–4.0)
KATE-2 (53) (NCT02924883)	Randomized phase II	Advanced	202	TDM1+atezolizumab/placebo T-DM1+placebo	• Median PFS (range) in the PD-L1+ population (exploratory analyses) • T-DM1+atezo:8.5 m • T-DM1+placebo: 4.1 m HR 0.60 (95%CI 0.32–1.11)
KATE3 (NCT04740918)	Randomized double-blind phase III	Advanced in PD-L1+ pts who have received prior trastuzumab+/− pertuzumab, and taxane	320	T-DM1+atezolizumab/placebo	Recruiting
NCT03125928	Single-arm phase II	1st line advanced	50	Paclitaxel+trastuzumab+pertuzum ab+atezolizumab	Recruiting
NRG-BR004 (NCT03199885)	Randomized double-blind phase III	1st line advanced	600	Paclitaxel+trastuzumab+pertuzumab with atezolizumab/placebo	Recruiting
SOLTI-ATREZZO (NCT04759248)	Single-arm phase II	Advanced PAM50 nonluminal/HER2+breast cancer refractory to trastuzumab/pertuzumab based therapy and T-DM1	110	Atezolizumab+trastuzumab+vinorelbine	Recruiting
Impassion050 (54) (NCT03726879)	Randomized double-blind phase III	Neoadjuvant	454	ddAC followed by paclitaxel+trastuzumab+pertuzumab with atezolizumab or placebo	• pCR in the PD-L1-positive population and ITT population • In the ITT population, pCR was 62.4% in the atezolizumab arm and 62.7% in the placebo (p=1.0) • In the PD-L1 positive population, pCR was achieved by 64.2% of the atezolizumab arm and 72.5% of the placebo arm (p=0.2)
KEYRICHED-1 (56) (NCT03988036)	Single-arm, phase II	Neoadjuvant	46	Pembrolizumab+trastuzumab and pertuzumab×12 weeks	• pCR in patients with PAM 50 HER2-enriched tumors In the per protocol population, pCR was 46% and 52% in all 46 evaluable patients
APTneo (NCT03595592)	Randomized, open-label phase III	Neoadjuvant	650	Pertuzumab and trastuzumab with carboplatin and paclitaxel+/− atezolizumab	EFS Recruiting
Astefania NCT04873362	Randomized, double-blind placebo-controlled phase III	Adjuvant	1700	Atezolizumab or placebo with T-DM1 in patients with residual invasive disease following preoperative therapy	IDFS Recruiting
NCT03632941	Randomized, phase II study	Advanced	39	VRP-HER2 Vaccination and Pembrolizumab	Recruiting

HR, hazard ratio; m, months; OR, objective response; ORR, overall response rate; PFS, progression-free survival; pts, patients; SD, stable disease; mets, metastases; pts patients; ddAC, dose-dense doxorubicin+cyclophosphamide; EFS, event-free survival; ITT, intention-to-treat; pCR, pathologic complete response; IDFS, invasive disease-free survival.

### 3.2 Cytotoxic T-lymphocyte antigen-4 (CTLA-4) inhibitor

CTLA-4 is a key inhibitory receptor expressed on effector T cells and regulatory T cells. After T cell activation, CTLA-4 signaling negatively regulates the immune response (59). Ipilimumab was the first anti- CTLA-4 agent approved by the FDA for the treatment of metastatic melanoma (59). Clinical trials in evaluating CTLA-4 inhibitors in HER2+ breast cancer are underway.

## 4 Adoptive T-cell immunotherapy

Adoptive T-cell immunotherapy involves re-infusion of autologous anti-tumor T lymphocytes to patients. Anti-tumor T cells are isolated from primary tumors or peripheral blood mononuclear cells (60). CAR-T technology is one type of adoptive T-cell immunotherapy. CAR-T by gene transfer to reprogram patients’ T cells to express chimeric antigen receptors (CARs) directs T cell cytotoxic potential against cancer cells. Anti-CD19 CAR⁃T cells have achieved a complete remission rate by 90% in B-cell acute lymphoblastic leukemia (61). Although CAR-T cell immunotherapy has promising efficacy in hematological diseases, few studies have been conducted in breast cancers, especially in HER2-+ breast cancers. T cells have better access to the tumor than antibodies and retain longer time *in vivo*. Thus, CAR T-cell therapies targeting the HER2 receptor were evaluated in preclinical and clinical settings. In pre-clinical models, dual-targeting CAR-T cells specific to co-expressing HER2 and MUC-1 effectively killed HER2+ breast cancer cells expressing these targets (62), HER2-redirected CAR-T cells eliminated HER2+ trastuzumab-sensitive tumor cell lines (SKBR3 and BT474). Similarly, a tri-specific antibody targeting HER2, CD3 and CD28 has caused regression of breast cancers in a humanized mouse model through CD4-dependent inhibition of tumor cell cycle progression (63–65). In 2010, the first reported HER2 CAR-T cell therapy resulted in a patient death due to the lower HER2 on lung epithelial cells (66). In order to reduce the off-tumor on-target effects, the HER2 specific CAR-T targeting different epitopes was designed. In recent years, many HER2 CAR T cell studies have been performed (67).Many HER2 CAR-T cell studies are ongoing or being analyzed, and we look forward to more results leading to more treatments. (**Table 2**).

**Table 2:** Clinical trials of HER2 CAR in breast cancer.

Clinical Trials.gov Identifier	Phase	Cell source	CAR signaling domain	Sponsor	Status
NCT02547961	Phase 1 Phase 2	Patient autologous T cells	Her2-, CD28-CD3ζ	Fuda Cancer Hospital, China	Withdrawn (Project terminated due to revision of local regulations)
NCT03696030	Phase 1	Patient autologous T cells	HER2(EQ)BBzeta/CD19t+	City of Hope Medical Center, US	Recruiting
NCT02713984	Phase 1 Phase 2	Patient autologous T cells	Her2-	Southwest Hospital, China	Withdrawn (Reform CAR structure due to safety consideration)
NCT04660929	Phase 1	Patient autologous T cells	Her2-	Carisma Therapeutics Inc, US	Recruiting
NCT04650451	Phase 1/2	Patient autologous T cells	Her2-	Bellicum Pharmaceuticals,US	Recruiting
NCT03740256	Phase 1	Patient autologous T cells	Her2-	Baylor College of Medicine, US	Recruiting
NCT04020575	Phase 1	Patient autologous T cells	MUC1*, huMNC2-CAR44	Minerva Biotechnologies Corporation,US	Recruiting
NCT04430595	Phase 1 Phase 2	Patient autologous T cells	Her2-, GD2- and CD44v6	Shenzhen Geno-Immune Medical Institute,China	Recruiting
NCT05007379		Patient autologous T cells		Centre Oscar Lambret, France	Not yet recruiting
NCT02792114	Phase 1	Patient autologous T cells	mesothelin	Memorial Sloan Kettering Cancer Center, US	Active
NCT04511871	Phase 1	Patient autologous T cells	CCT303-406	Shanghai PerHum Therapeutics Co., Ltd.	Recruiting
NCT04842812	Phase 1	Patient autologous T cells	HER2, Mesothelin, PSCA, MUC1, Lewis-Y, GPC3, AXL, EGFR, Claudin18.2/6, ROR1, GD1, or B7-H3	Second Affiliated Hospital of Guangzhou Medical University, US	Recruiting
NCT04025216	Phase 1	Patient autologous T cells	TnMUC1	Tmunity Therapeutics, PA	Active

Data obtained from clinicaltrials.gov.

## 5 Active immunotherapy

Active immunization, such as vaccination, refers to the use of antigenic stimulation to allow the body to attack cancer cells. Cancer vaccines as active immunotherapyare designed to activate autologous immune cells to elicit a serial of anti-tumor immune responses to achieve therapeutic goal (68, 69). In breast cancer, immunization of peptides derived from tumor –associated antigens represents the most common vaccination approach. Cancer protein and carbohydrate antigens have also been explored as vaccines. Tumor cells as vaccines are traditional methods, while DNA-based and dendritic cell (DC)-based vaccines represent novel modalities in this field (70).

### 5.1 Polypeptide vaccines

Construction of polypeptide vaccines is relatively simple. Polypeptide fragments derived from tumor associated antigens are easy to be produced and purified in large quantities. Polypeptide vaccines exert their immunogenicity by binding to antigen-presenting cells (APC) or T cells to inducecellular and humoral immunity. One of the most commonly used therapeutic strategies for breast cancer is to activate the immune response to specific tumor antigens by delivering MHC class I restricted peptide epitopes. Compared to other formulations, the production of short amino acid polypeptides is simple, the cost is low, and the transportation is relatively stable, making large-scale production and transportation possible. However, a single peptide is usually limited to some human leukocyte antigen (HLA) subtypes, so patients who do not express common HLA types cannot be treated with this vaccine. In addition, the usual MHC class I binding peptide has no strong ability to activate CD4 helper T cells, which may lead to limited activation of CD8 cytotoxic T cells and transient immune response. This problem can be partially overcome by using synthetic peptides of sufficient length to include multiple MHC class I and class II epitopes.

The magnitude and durability of the immune response generated by polypeptide vaccines can be enhanced by increasing the size through conjugation of the polypeptides to carrier proteins or by the addition of immunologic adjuvants. Specifically, granulocyte macrophage clonal stimulating factor (GM-CSF) which canpromote antigen presentation and enhance T-cell responses has the potential of enhancing vaccine efficacy. In breast cancer, HER2 peptide vaccines include E75 (HER2 369-377), GP2 (HER2 654-662) and AE37 (Ii-Key/HER2 776 -790hybrid) peptides. E75(from the extracellular domain) is a 9 amino acid peptide derived from the extracellular domain of HER2. E75 can bind to human leukocyte antigen-A2 (HLA-A2) and HLA-A3 on APCs, and stimulate cytotoxic T lymphocytes (71). In an early phase clinical trial in node-positive and high-risk node-negative breast cancer patients, E75 was immunogenic, 5-year disease-free survival rates were 89.7% and 80.2% (P = 0.08) in the vaccinated vs. control groups, respectively, and relative risk of recurrence was reduced by 48% (72). GP2(transmembrane domain) is a 9-amino acid major histocompatibility complex (MHC) class I peptide derived from the transmembrane domain of HER-2 (73). In a prospective, randomized, single blind, multicenter study of disease-free patients with HER2+, node positive, or node negative breast cancer after treatment with standard of care, GP2 and AE37(intracellular domain) were safe and able to improve disease-free survival in select patients. In a prospective, randomized, placebo-controlled, single blind phase IIB, multicenter clinical study, patients with operable HER2 3+ breast cancer treated with GP2 + GM-CSF after trastuzumab had a PFS rate of 100% after 5 years of follow-up compared to 89.4% in patients treated with GM-CSF alone. Liposome modification technology can improve immunno-efficacy of Peptide Vaccines. More studies have shown that APCs readily take the immunogenic molecules from the displaying phage, process and present them on MHC I and MHC II molecules to induce higher immune responses compared with soluble antigens with no carriers (74).A particularly effective immune strategy to induce T-cell responses against tumor antigens is to use DCs loaded with the tumor antigen.It is feasible and safe to generate and administer HER2-loaded DCs to patients with advanced HER2/neu-expressing malignancies and high-risk breast cancer (75).

### 5.2 Protein-based vaccines

To expand the immune response to different epitopes of the HER2 protein, researchers have developed vaccines composed of intact or truncated HER2 (such as the intracellular or extracellular domain) (76). MUC-1 is a tumor-associated antigen that is glycosylated in tumor cells. In a phase III trial, vaccination of breast cancer patients with oxidized mannan-MUC1 resulted in significantly lower recurrence rates than placebo (77). In a phase 1 trial, administration of a protein-based vaccine derived from amino acids 676-1255 of the intracellular domain of HER2 plus GM-CSF resulted in HER2-specific T cell and antibody immunity in patients with stage II-IV HER2+ breast and ovarian cancer (78). Cellular immunity was maintained approximately one year after completion of immunization in >50% of patients.

In another phase 1 trial, there was an association between vaccination dose, the immunization schedule, and the prevalence of HER2-specific humoral responses after administration of recombinant HER2 protein with adjuvant AS15 in trastuzumab-naive patients with stage II-III HER2+ breast cancer that had undergone surgical resection and adjuvant therapy (79). HER2-specific immunity was maintained > 5 years in the majority of patients that received the highest dose of vaccine (80).

### 5.3 DNA-based vaccines

DNA vaccines comprise a bacterial plasmid encoding the antigen of interest, which is produced *in vivo* to elicit an immune response. As a breakthrough technology, the DNA vaccine was selected in the list of top ten emerging technologies in the world in 2017. DNA is stable and easy to clone, which makes it easy to produce and preserve. In a phase 1 trial, patients with metastatic HER2+ breast cancer treated with trastuzumab received a DNA vaccine encoding a full-length signaling-deficient version of HER2, plus GM-CSF and IL-2 (81). Two patients discontinued treatment due to disease progression or disease-related complications. The vaccine was considered safe. There was a significant increase in T cell responses to HER2 at long-term follow-up.

In a multicenter phase 1 trial, the DNA vaccines V930 or V930 followed by V932, were well tolerated in patients with stage II-IV solid tumors expressing HER2 or carcinoembryonic antigen (CEA). V930 and V932 expressed the extracellular and transmembrane domains of HER2 and CEA. No measurable cell-mediated immune response to HER2or CEA was detected (82). A clinical trial of a DNA vaccine based on the HER2 intracellular domain(ICD) plasmid to evaluate the safety and immunogenicity of this HER2-targeting breast cancer vaccine. A total of 66 patients with stage III and IV HER2-positive breast cancer were included in the study, of which 65 completed 3 vaccinations. The researchers found that the use of trastuzumab at the same time as vaccination was associated with the persistence of the HER2 vaccine. During the 10-year follow-up period, median OS and PFS had not been achieved in any group as of press time. In the intermediate-dose group, 100% of stage III patients were still alive at the time of last follow-up (83).

### 5.4 Dendritic cell (DC)-based vaccines

DCs are a population of APCs that process antigens into polypeptide fragments, present them to naive T cells, and generate CD4 helper T cells and CD8 T cell-mediated cellular immunity. DCs can also induce humoral immunity, activating NK cells and NKT cells (84). DC-based vaccines are constructed from DCs extracted from patients that are exposed to digested tumor peptides or messenger RNA from the patient’s autologous tumor. DCs express high levels of HLA I and II molecules and costimulatory protein, and produce cytokines necessary for T cell activation, thereby stimulating naive and memory T cells. Patients with HER2+ intraductal carcinoma vaccinated before surgical resection with DCs pulsed with HER-2/neu HLA class I and II peptides and activated *in vitro* with IFN-gamma and bacterial lipopolysaccharide, showed accumulation of T lymphocytes and B lymphocytes in the breast, and induction of complement-dependent, tumor-lytic antibodies. HER-2/neu expression was decreased in surgical tumor specimens (85). In a preclinical mouse model of HER2^+^ breast cancer, class II HER2 peptide pulsed DC1 (Class II HER2-DC1) drove CD4+ and CD8+ T cells infiltrating into tumors and delayed tumor growth. It also demonstrated that Class II HER2-DC1 in combination with anti-HER2 therapy and checkpoint blockade improved survival (86).

### 5.5 Tumor cell vaccine

Tumor cell vaccines are characterized by using whole tumor cells or products of tumor cell lysis to elicit an immune response. Autologous whole tumor cells can self-stimulate an immune response or provide a pool of antigens for APC presentation (87). Autologous whole tumor cell vaccines generate polyclonal adaptive immune responses that are only applied to the specific individual patient. In patients with metastatic breast cancer, the combination of the whole tumor cell vaccine GV-AX with low-dose cyclophosphamide and doxorubicin elicited an effective immune response (88). In patients with HER2+ metastatic breast cancer, GV-AX in combination with trastuzumab and low-dose cyclophosphamide was associated with gradual expansion of multifunctional HER-2-specific T cells throughout the vaccination cycle (89).

### 5.6 Viral vector-based vaccines

Viruses are naturally immunogenic, and their genetic material can be engineered to carry any transgenes to be expressed in host cells. Some recombinant viruses are able to infect and express transgenes in immune cells such as antigen-presenting cells. In addition, some viruses have natural oncolytic properties or can be engineered to target tumor cells (oncolytic virus therapy). Oncolytic viral therapy has the added advantage of eliciting immune T cell responses to viral antigens as well as tumor cell antigens.

At present, the most commonly used viral vectors are lentivirus (LV) vectors, adenovirus (AdV) vectors and adeno-associated virus vectors, and the latter two are mostly used in the field of cancer treatment.

Hartman et. used a novel viral vaccine vector to elicit HER2-specific adaptive immune responses. This study showed: Vaccines that target tumor antigens will not be as effective as targeting true oncogenic drivers, and neither stimulating tumor-specific T cells nor blocking key immune checkpoints is sufficient to overcome the immunosuppressive layer (90).

Single-stranded RNA viruses are used in self-replicating vaccine platforms, including positive-stranded viruses (alphaviruses and flaviviruses) and negative-stranded viruses (measles viruses and rhabdomyoviruses) (91).Studies have shown that srRNA vaccine platforms (including those using bare RNA, DNA-emitted replicons, viral replicon particles (VRPs), and, more recently, synthetic srRNA replicon particles, are potent inducers of anti-tumor immunity that can be enhanced by homologous vaccine enhancement and in combination with chemotherapy, radiation, and immune checkpoint inhibition (92). Sourabh Shukla et al. evaluated plant virus nanoparticle (VNP) platform technology for the treatment and prevention of HER2+ malignancies (93). Lyerly and colleagues constructed a vaccine that uses a neutral viral vector to carry genetic information against the HER2 protein. Once in the body, the vaccine targets the HER2 protein in cancer cells, which triggers the immune system to attack the cancer. In preclinical studies, mice were implanted with HER2+ tumor cells and treated with VRP-HER2 vaccine, and HER2-specific immune response and antitumor function were assessed. Results: Vaccination induced the production of HER2-specific T cells and antibodies while inhibiting tumor growth. The tumors in the mice regressed. A subsequent Phase I clinical trial enrolled 22 breast cancer patients with advanced HER2+ tested for VRP-HER2, and one group received VRP-HER2 every 2 weeks for a total of three doses. VRP-HER2 vaccine increases HER2-specific memory CD8 T cells and has antitumor effects in preclinical and clinical studies (94). Researchers at Duke University are currently initiating a phase II clinical trial (NCT03632941) combining VRP-HER2 with PD-1 (pembrolizumab) in advanced HER2+ BC. (modified vaccinia Ankara) MVA-BN-Brachyury/(fowlpox virus)FPV-BN-brachyury vaccine is well tolerated and induces immune responses to brachyury and cascade antigens and demonstrates some evidence of clinical benefit in solid tumors (95).In a study by Gao et al., a combination of recombinant oncolytic virus with CTLA-4 antibodies targeted HER2 breast cancer cells. This therapy can cure most mice, while viral therapy alone can only prolong survival (96).

### 5.7 Combination of vaccines with immune checkpoint inhibitors

AD vector vaccines encoding tumor neoantigens in combination with PD-1 inhibitors have a good tumor suppression effect in mouse models (97). However, the mechanism of the combination therapy for cancers has not been elucidated. Recently, researchers from the Italian Institute of Genomic Medicine and the Candiolo Cancer Institute, have found that the combination of gorilla adenovirus (GAd) vaccine against tumor neoantigens and PD-1 inhibitors can enhance the stemness of neoantigen-specific CD8+ T cells, increase their number, improve immunogenicity, and thus enhance anti-tumor efficacy (98). An increased proportion of Tcf1+CD8+ T cells in immune checkpoint inhibitor (ICI) therapy has been positively correlated with a longer duration of response to ICI therapy (99). Therefore, maintaining the differentiation of Tcf1+CD8+ T cells plays an important role in improving the efficacy of immunotherapy, increasing the number of Tcf1+CD8+ T cells, and maintaining their self-renewal ability, which is expected to overcome the problem of treatment resistance encountered by ICI (100). Meanwhile, multiple studies have shown that the clinical efficacy of PD-1 inhibitors depends on the quantity, quality, and tumor invasion characteristics of CD8+ T cells targeting mutation-associated tumor neoantigens (101, 102). As a result, neoantigens have become targets for cancer vaccines.

In summary, the combination of AD vector vaccine and PD-1 inhibitor can improve the stemness of tumor neoantigen-specific CD8+ T cells, maintain and promote their ability to proliferate, expand and transport to tumor sites, and promote and induce memory T cells to produce an immune response against tumors.

## 6 Conclusion and perspective

Breast cancer has multiple molecular subtypes and comprehensive therapeutic approaches are needed to treat this disease. Immunotherapy is emerging as a promising therapeutic strategy for HER2+ breast cancer, and several clinical trials are ongoing. Here, we summarized recent advances in immunotherapy for HER-2+ breast cancer, in the areas of passive immunotherapy, immune checkpoint inhibitors, adoptive T cell immunotherapy, and active immunotherapy. These include therapies that activate and enhance ADCC activity against HER2+ breast cancer cells or enhance the activity of effector T cells (immune checkpoint inhibitors), often in combination with existing anti-HER2-targeted regimens; adoptive T cell immunotherapy; and the development of tumor vaccines. Immunotherapy has a good application status and prospect in HER-2+ breast cancer, and it is expected that more trial results will better guide clinical application.

## Author contributions

TY, structure and drafting the original manuscript; LK, review idea; TY and LK writing conclusion and perspective section; TY, LK, DL, and YS, revised and approved the final manuscript. All authors contributed to the article and approved the submitted version.
